# Investigating the Effect of Cognitive–Behavioral, Mindful-Based, Emotional-Based Intervention and Professional Training on Teachers’ Job Burnout: A Meta-Analysis

**DOI:** 10.3390/bs13100803

**Published:** 2023-09-27

**Authors:** Jian Li, Eryong Xue, Yunshu He

**Affiliations:** China Institute of Education Policy, Faculty of Education, Beijing Normal University, Beijing 100875, China; jianli209@bnu.edu.cn (J.L.);

**Keywords:** teachers’ job burnout, interventions, meta-analysis, cognitive–behavioral intervention (CBI), mindfulness-based intervention (MBI)

## Abstract

Teachers are more likely to experience occupational burnout; intervention for their job burnout has been paid more and more attention by the academic community. However, there is not enough evidence to support the interventions’ effect. This study adopts the meta-analysis method and makes a statistical analysis of the interventions’ effect on teachers’ job burnout based on 29 papers in the literature of randomized controlled experiments. It mainly presents the basic external characteristics of the research literature; besides, based on the results of software CMA.V3, this paper also analysed the overall intervention effect and the separate effect of four types of intervention, namely, cognitive–behavioral interventions (CBI), mindfulness-based interventions (MBI), professional training (PT), and emotional-based intervention (EBI). The results showed that CBI had the best effect, and mindfulness-based intervention had the second-best effect. Professional training also showed a good intervention effect, while the intervention effect of emotional-based intervention remains to be verified. In addition, it was found that only the effect of cognitive–behavioral intervention (CBI) was significant and had a strong effect (g = 0.876, 95% CI-1.06, 2.814, *p* < 0.05) when considering their effects on emotional exhaustion, while the other three interventions had no significant effect. The discussion section is provided last.

## 1. Introduction

In 1974, American clinical psychologist Herbert J. Freudenberger first proposed the concept of “job burnout”, which refers to the extremely physical and emotional fatigue caused by overwork [1]. The frequently used conceptual framework for burnout is the three components framework proposed by Masrazzi and his colleagues which identified three factors: emotional exhaustion, depersonalization, and low personal accomplishment. This also forms the Masrazzi burnout scale, which is now widely used to measure job burnout. Emotional exhaustion is when a person often feels lack of energy and exhausted emotional resources. Depersonalization refers to considering the customer as an object rather than a person. Employees may appear detached and emotionally callous, and may be suspicious of colleagues, customers, and organizations. Low personal accomplishment mainly means that employees are characterized by negative self-evaluation and reduced self-efficacy, that is, feeling inadequate about their personal or professional abilities. Individuals have a reduced sense of competence and achievement at work or in interactions with others [2,3].

Ferres divided the stages of teachers’ career development into pre-service preparation stage, entry stage, formation stage, enthusiasm and growth stage, career frustration stage, stability and stagnation stage, career discharge stage, and career-end stage. Teacher burnout may occur in the stages of the latter half of their careers. In fact, with developments of the current times, the importance of education is self-evident, and teachers are facing higher requirements for various aspects. For example, Roeser et al. (2013) [4] found that current teachers encountered psychological, physiological, and occupational stress and burnout. This means that teachers can experience temporary or persistent burnout at any stage throughout their career. There are many reasons for teacher burnout, mainly related to teachers’ work and their personal lives. At work, when teachers fail to obtain adequate organizational support, a suitable working environment, reasonable salary, and good relationships with colleagues, they will suffer from a high level of psychological burnout [5,6,7]. In addition, teachers’ individual psychological characteristics (such as resilience, mindfulness, and neuroticism), work ability, work experience [8], age, gender, etc., will also determine the level of teacher burnout [9,10,11]. Mild job burnout may cause teachers’ low work efficiency [12], while serious job burnout will not only increase teachers’ turnover intention, but also cause psychological and physiological conditions, such as depression and cardiovascular diseases [13,14].

Many schools and education authorities have taken measures to reduce teachers’ burnout and increase their happiness at work; besides, many scholars have already conducted research on intervention into teacher burnout. This study adopts the meta-analysis method and makes a statistical analysis of the interventions’ effect on teachers’ job burnout based on 29 samples of literature of randomized controlled experiments. It mainly presents the basic external characteristics of the research literature. The current types of intervention are mainly cognitive–behavioral intervention (CBI), mindfulness-based intervention (MBI) [15], and emotion-based intervention (EBI). At the same time, there is also a type of intervention which aims to improve professional skills in education and teaching, and to promote skills in communication with others, thus, to reduce the burnout syndrome of teachers [16]. Knowledge-based interventions (KBI) have also been used in teacher training in the past. But because they usually do not include practical steps, KBI is usually embedded in other types of intervention, or is not given priority [17]. Therefore, KBI-related research will not be introduced below.

## 2. Literature Review of Cognitive–Behavioral, Mindful-Based, and Emotional-Based Interventions and Professional Training on Teachers’ Job Burnout

### 2.1. Cognitive–Behavioral Interventions (CBI)

CBI is not one specific method or set of steps, which usually consists of different methods. It mainly combines cognitive training and strategic behavioral practices to provide teachers with the knowledge and skills needed to manage work-related stress. And CBI normally have an excellent effect on teachers’ job burnout, according to the existing research. Ghasemi (2023) [18] used a randomized controlled experiment to investigate the effect of group-based cognitive–behavioral intervention on 242 English teachers. The results of the Masrazzi burnout table showed that the intervention group experienced significantly greater improvements in burnout. And another Ghasemi [19] study also proved the results, which additionally indicated that the effect of CBI even became more significant in the half-year follow-up assessment. While CBI is sometimes not used alone, Dike et al. (2021) [20] took 58 teachers with autistic children as subjects to explore the effect of Yoga-CBT (Y-CBI) intervention on job burnout. The results showed that the Y-CBI group had significantly lower job burnout in the post-test assessment compared to the candidate group. At the three-month follow-up assessment, the reduction in participants’ burnout was sustained. Cheek et al. (2003) [21] found that when CBI and music intervention techniques were combined, teachers had lower levels of burnout symptoms than when CBI was used alone.

### 2.2. Mindfulness-Based Interventions (MBI) and Other Relaxed Therapy

Mindfulness employs cognitive and behavioral strategies that focus on the process of feeling and thinking, rather than the content of thinking [22]. Awareness and acceptance without judgment are key components of a mindfulness strategy [23]. Mindfulness practices are often performed in a variety of ways, such as static meditation, breathing exercises, dynamic yoga exercises, and other relaxing methods. And in most studies, MBI produces a good effect on teachers’ burnout. Cheng et al. (2022) [24] used a hybrid approach designed to assess the feasibility and effectiveness of a mindfulness-based adaptive program for kindergarten teachers. The results showed that, compared to the control group, the mindfulness training group showed significant improvements in emotional intelligence and mindfulness in teaching, and reductions in burnout and depression. Roeser et al. (2013) [4] investigated the effects of randomization to mindfulness training (MT) or waiting-list control conditions on psychological and physiological indicators of teacher occupational stress and burnout. The results showed that 87% of teachers completed the program and found it beneficial. Compared with the control group, teachers randomly assigned to MT demonstrated greater mindfulness, concentration, and working memory abilities, as well as occupational self-compassion, and lower levels of occupational stress and burnout after the program and at follow-up. In the Internet age, many applications for mindfulness technology have been developed. Russell et al. (2013) [25] explored the effectiveness of mindfulness-based smartphone app interventions in reducing burnout among special educators. The intervention level of the experimental group decreased significantly after intervention. However, at the follow-up two weeks later, there was no significant increase in mindfulness scores, which means that the mechanism of this reduction in personal burnout still has considerable room for research.

### 2.3. Professional Training (PT)

Providing teachers with professional training, such as teaching training, classroom management training, training for communicating with students and colleagues, and self-psychological adjustment training will help teachers to manage the classroom and deal with various challenges from teaching and schoolwork, thus reducing the probability of teachers suffering from burnout. Rivas et al. (2023) [26] measured the changes of teachers’ self-efficacy and job burnout after participating in teacher–child interactive training (TCIT) through a randomized wait-list controlled study, and the results showed that TCIT is an effective intervention. It can improve teachers’ efficacy in classroom management, teaching strategies, and student engagement, and reduce burnout levels. Sandilos et al. (2020) [27] proved that when teachers participate in children’s social–emotional learning training programs, they will have better interaction with students, and their own job burnout level will decline, while teachers in the control group will have higher job burnout level. Allen et al. (2019) [28] evaluated the role of teacher classroom management training (TCM) in enhancing teacher well-being, reducing burnout, and improving self-efficacy.

### 2.4. The Emotional-Based Interventions (EBI)

Emotional-based intervention is often conducted at the same time as other cognitive and behavioral interventions [29], and most of them occur as part of clinical psychological intervention. Although there are many programs on teacher socio–emotional skills training, this type of intervention is classified in this paper as a type of professional training (PT). Capaciar International is an emotion-oriented intervention program developed by Cane that is widely used primarily to achieve emotional integrity and well-being in people with burnout [29]. In addition, physical energy exercises and emotional freedom techniques have been used clinically to reduce stress and burnout, but relevant academic research has been limited. There are also more niche intervention techniques. Chan (2011) [30] investigated the effectiveness of gratitude intervention programs in increasing life satisfaction and reducing symptoms of burnout. In the post-intervention assessment, an increase in life satisfaction and personal fulfillment and a decrease in emotional exhaustion and depersonalization were observed. The results showed that teachers’ sense of control over their work is significantly increased, teachers’ job burnout is moderately reduced, and their stress is reduced [31].

To sum up, most of the research results show that intervention has a good effect on teacher burnout, but there are a few studies that do not show a significant effect. In addition, different intervention methods have different effects on teacher burnout. The following questions are raised in this study:

Q1: Does intervention influence teacher burnout?

Q2: What is the effect of different intervention types on teachers’ burnout intervention? Is there any difference?

## 3. Method

In this paper, the meta-analysis methods are adopted to conduct a comprehensive and scientific analysis of the research focusing on the interventions’ effect on teachers’ burnout. Themeta-analysis methods refer to the collection and analysis of literature in a specific field in multiple databases according to certain standards and principles, which can sort out knowledge in a specific field, identify existing problems in current research and future research directions, and even draw new theories. In the meta-analysis research part of this paper, the software Comprehensive Meta-analysis V3 is mainly used for the effect’s analysis [32].

### Data Resources

In this study, Web of Science, Proquest, and Eric were used as literature retrieval databases. “Teacher or educator or instructor or school staff or faculty” and “burnout OR exhaustion OR depersonalization OR inefficacy “and ”intervention or treatment or therapy or program“ were the search terms to obtain the preliminary literature. Then, by using the filtering function of the Web of Science database, the literature type is limited to article, core database source, English, and 1949 literature was obtained. On Proquest, the literature was limited to full-text literature, peer review, academic journal literature, and English. Finally, 1120 pieces of literature were obtained. On Eric, the literature was limited to full-text literature, peer review, references, academic journal literature, and English, and 241 articles were obtained. After a preliminary search, a total of 3310 articles were obtained.

**Figure 1:**
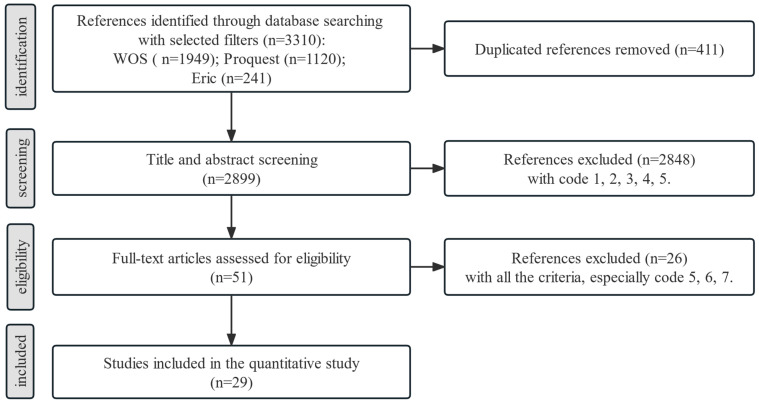
Process of publication exclusion.

Formal literature retrieval is divided into three steps, which have to be processed according to the exclusion criterion in Table 1. The specific screening process is shown in Figure 1. Step 1 is to screen out duplicates. Step 2 is to review the title and abstract to exclude literature according to the first 5 Codes in Table 1. Step 3 is to browse the full text to exclude the literature according to all the criteria. For step 3, the aim is to exclude the literature omitted in step 2, to exclude the literature that use one-group experiments or do not report relevant statistical values. Finally, 29 pieces of literature were obtained.

## 4. Results

After strict screening, this paper finally obtained 29 pieces of literature that met the standards to explore the effect of teacher burnout intervention, which mainly had the following basic characteristics (Table 2). From the perspective of publication years, the number of research documents in this paper shows an overall increasing trend, with a large increase in recent years, indicating that the academic community has paid more and more attention to the intervention effect of teacher burnout in recent years. In addition, literature research covers 13 countries, including Nigeria, the United States, China, and Germany. At the same time, the objects of this study covered the whole vertical education system, but mainly concentrated on the basic education stage (*n* = 15), and there were few studies which discussed the interventions’ effect on teachers’ burnout in preschool education and higher education stages. Finally, in terms of some special characteristics of the research objectives, special education teachers are the object of the researchers’ focus.

The measurement of teacher burnout in the research literature in this paper mainly uses aspects of Marzari’s burnout scale, which are mainly concerned with emotional exhaustion, depersonalization, and personal achievement. And the related questionnaires, such as the Spanish burnout inventory (SBI) [33] and Dutch-MBI [34], are also widely used. There are also a small number of other questionnaires, such as the teacher burnout inventory (TBI) [35] and the Copenh burnout inventory (CBI) [36]. Additionally, there are four main types of intervention used in the literature selected for this paper. Based on analysis of the introduction sections, the four types are cognitive–behavioral intervention (CBI), mindfulness behavior intervention (MBI), professional training (PT), and emotional-based intervention (EBI). Among them, MBI and CBI are often used, and PT is sometimes used to reduce teacher burnout. Most of the research literature in this paper uses one intervention mode alone, while a few studies use two or more intervention modes, such as yoga-based CBI [37], and MBI and PT combined [38].

**Table 2:** Basic features of the publication.

Item		Sub-Item	Example Studies
Publication Year		2003 (*n* = 1), 2011 (*n* = 2), 2013 (*n* = 2), 2014 (*n* = 1), 2015 (*n* = 1), 2017 (*n* = 1), 2018 (*n* = 2), 2019 (*n* = 3), 2020 (*n* = 3), 2021 (*n* = 8), 2022 (*n* = 5)	\
Participants’ characteristics	Country	Nigeria (*n* = 5), USA (*n* = 4), China (*n* = 3), Germany (*n* = 3), Iran (*n* = 2), Canada (*n* = 2), Italy (*n* = 2), Israel (*n* = 2), Ireland (*n* = 1), UK (*n* = 1), Spain (*n* = 1), The Netherlands (*n* = 1), South Africa (*n* = 1)	\
	School level	pre-school (*n* = 1), primary school (*n* = 9), secondary school (*n* = 6), university (*n* = 1)	\
	Others	special education (*n* = 5), English teacher (*n* = 1), Class tutor (*n* = 1), rural school (*n* = 1)	\
Burnout measure		MBI-ES (Maslach et al., 1996), MBI-GS (Maslach et al., 1986) (*n* = 22)	Cheng et al., 2021 [24]; Latino et al., 2021 [39]
		SBI (Gil-Monte, 2011) (*n* = 1)	Shi and Ruckthum, 2022 [40]
		D-MBI (Schaufeli and Van Dierendonck, 2000) (*n* = 1)	Ugwoke et al., 2018 [41]
		TBI (*n* = 1)	Nwabuko et al., 2019 [35]
		CBI (Kristensen et al., 2005) (*n* = 1)	Russell et al., 2022 [25]
		PQOLS-Burnout (Stamm, 2010) (*n* = 1)	Ibigbami et al., 2021 [42]
		BAT-Burnout (Schaufeli et al., 2020) (*n* = 1)	Ogakwu et al., 2022 [43]
		Modified mini-z survey—Burnout (*n* = 1)	Ghasemi et al., 2022 [18]
intervention-types		Cognitive–behavioral intervention (CBI) (*n* = 11)	Ogakwu et al., 2021 [43]; Schnaider-Levi et al., 2020 [44]
		Mindfulness behavior Intervention (MBI) (*n* = 13)	Russell et al., 2022 [25]; Latino et al., 2021 [39]
		Professional training (PT) (*n* = 6)	Allen et al., 2019 [28]; Shi and Ruckthum et al., 2022 [40]
		Emotional behavior intervention (EBI) (*n* = 3)	Ugwoke et al., 2018 [41]
		others (*n* = 3)	Ebert, 2014 [45]

Note: PQOLS-Professional Quality of Life Scale; BAT-Burnout Assessment Tool.

### 4.1. Effects of the Intervention on Burnout

This part presents the overall effect of intervention on teacher burnout, and the effect of the four types of intervention on teacher burnout are also presented. It should be noted that most of the research literature in this paper uses the Masrazzi burnout scale. Therefore, the three dimensions of the Masrazzi burnout scale are taken as the statistical standard in this paper, which may affect the accuracy of the results to some extent. In addition, for the literature that did not report overall burnout, the average of reported dimensions was considered when calculating overall burnout. Another detail that must be explained in this part is that the study of Johnson and Naidoo’s (2017) [29] uses three different specific methods which are the same type of intervention, and the results were counted three time; thus, there was a total of 31 results in the end, rather than 29 results.

### 4.2. Overall Effects

**Figure 2:**
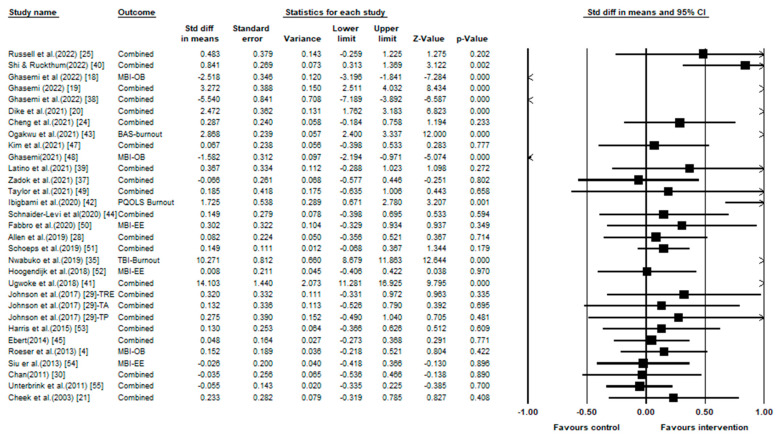
Standardized effect sizes and forest plot for the entire sample of studies for overall burnout symptoms. Note: in the outcome column, “combined” means the average effect, MBI-OB means the study report the overall burnout. And others, such as MBI-EE means the study only report the single dimension of burnout, emotional dimension [4,18,19,20,21,24,25,28,29,35,37,38,39,40,41,42,43,44,45,47,48,49,50,51,52,53,54,55].

Due to the mixed characteristics of the literature referred to in this paper, the random effects model is preliminarily used for meta-analysis. In Figure 2, the effect of intervention on overall burnout of each study are presented. In the right graphic display area, the black square represents the weight of the study. The larger the square, the greater the weight. The center point of the square represents the point estimate. The length of the horizontal line shows the confidence interval of the study. The arrow indicates that the 95% confidence interval of the effect size exceeds the display range. The direction of the arrow indicates the direction of the supportive results. Therefore, seen from fig.2, the results of almost 2/3 studies favor the intervention. In a classified way, the overall burnout symptoms using the scale were reported in 13 of 29 articles; emotional exhaustion was reported in 21 pieces of literature, depersonalization was included in 13 papers, and personal accomplishment was included in 16 papers (see Figure 2 and Table 3). If the effect size was between 0.2 and 0.6, the effect was moderate. If the effect value was greater than 0.6, it was a strong effect [46]. As a result, the effect size of interventions on overall burnout and the average effects of interventions on the separate dimensions were all significant. The strongest mean effect size was regarding depersonalization (g = 0.747; 95% CI0.259, 1.234; *p* = 0.003), and close to it was the average effect size for overall burnout (g = 0.644; 95% CI0.22, 1.068; *p* = 0). Interventions on emotional exhaustion showed an average effect of moderate strength (g = 0.387; 95% CI 0.04, 0.809; *p* = 0); while the effect size of intervention on personal accomplishment (g = 0.147; 95% CI 0.42, 0.712; *p* = 0) presents a weak average effect.

The heterogeneity test results of this paper can also be obtained from Table 3. The results indicate that both the overall burnout and the other three component dimensions have Q values greater than degrees of freedom and *p* values less than 0.01, indicating heterogeneity existed among sample effect values. This study uses I^2^ statistics as the basis for determining the level of heterogeneity, that is, I^2^ < 25% (low), 25% ≤ I^2^ ≤ 75% (medium), and I^2^ > 75% (high) indicate different degrees of heterogeneity [56]. The I^2^ values in this study were all higher than 75%, indicating that the heterogeneity of sample effect values was high, which provided a theory basis for selecting a random effects model for analysis.

### 4.3. Effectiveness for Different Intervention Approaches

Meta-analysis is conducted with the software Comprehensive Meta-Analysis 3.0 (CMA 3.0) (Biostat, Englewood, NJ, USA). CMA 3.0 is a flexible meta-analysis software that provides multiple calculation templates, which can be selected for analysis based on the type of trial and the type of data provided in the objective studies. The 29 studies selected in this article are controlled pre and post test (which can be selected in the software), and they all provided the data including mean, standard deviation, and sample size in the two groups of pre–post test (corresponding options are also provided in the software). After selection, the software created a header based on the options, including literature name, intervention type, result type, statistics, and effect quantity. Then, according to the table header, extracted and organized the data from 29 pieces of literature into an Excel table, and listed the data provided in each literature on the different dimensions of the impact of different types of interventions on burnout. Then, it copied the data Into CMA 3.0, which automatically calculated various effects, statistics, etc. There was a prerequisite step here, which was to extract and classify the specific intervention methods used in each piece of literature into four types of interventions. Due to the need to categorize and discuss the effects of different types of interventions on different dimensions of burnout, CMA 3.0 provided corresponding options to choose the types of intervention and burnout dimensions. According to research needs, the results can be presented in a permutation and combination manner. Because all the data used for calculation and the results to be presented use the same template, the outcomes were comparable. In this part, the average effects of different types of intervention on the single components of job burnout are discussed comparatively in the first paragraph below. And the effect of single-type interventions on the three dimensions of burnout are also analyzed separately in the second paragraph below.

As mentioned above, this paper divides intervention into four types. In terms of the effect of several interventions on emotional exhaustion, only the CBI effect was significant and had a strong effect (g = 0.876, 95% CI-1.06, 2.814, *p* < 0.05), while the other three interventions had no significant effect. In addition, except that no literature has discussed the effect of EBI on depersonalization, the other three types have significant effects on depersonalization: CBI (g) = 2.063, 95% CI 0.565, 3.562, *p* < 0.05), MBI (g) = 0.632, 95% CI 0.047, 1.218, *p* < 0.05), and PT (g = 0.225, 95% CI 0.26, 0.711, *p* < 0.01). CBI has the strongest effect on depersonalization, MBI has a stronger effect on depersonalization, and PT has a weaker effect on depersonalization. Finally, CBI, MBI, and PT have a significant effect on personal accomplishment. MBI (g = 0.479, 95% CI-0.17, 1.13, *p* < 0.01) and PT (g = 0.14, 95% CI-0.24, 0.522, *p* < 0.05) showed positive and moderate to weak effects, while the effect of CBI on personal accomplishment is negative and the average effect is small (See Table 4).

In terms of the intervention effect of CBI, the impacts of CBI on emotional exhaustion, depersonalization, and personal achievement are all significant, and the intervention effect on depersonalization is the largest. The impact on emotional exhaustion was second and the impact on personal achievement was negative. The results of the impact on depersonalization are consistent with the previous results of the overall effect, and the overall intervention has the largest impact on depersonalization. The most pieces of literature use MBI as the intervention method. MBI has a significant effect on depersonalization, and personal achievement effect, but had no significant effect on exhaustion. Personal training has a significant impact on depersonalization and personal achievement, but the effect is weak. And there is no significant impact on emotional exhaustion. There is no literature related to the effects of EBI on depersonalization, and its effects on emotional exhaustion and personal achievement are not significant.

### 4.4. Publication Bias

**Figure 3:**
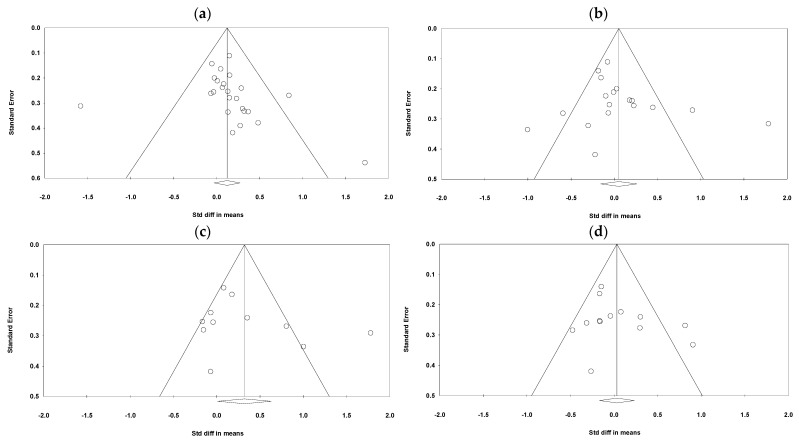
Funnel plots for publication bias.

This article starts with a funnel plot that includes overall burnout (*n* = 31), emotional exhaustion (*n* = 21), depersonalization (*n* = 13), and exhaustion (*n* = 31). The pieces of literature dealing with personal accomplishment (*n* = 16) was subjected to a publication bias test. The effect size is taken as the horizontal co-ordinate and the standard error as the vertical co-ordinate. The larger the sample size, the smaller the standard error, the higher the accuracy, leading to the more concentrated research distribution in the middle and upper part of the graph. The sample size is smaller, the standard error is larger, the accuracy is lower, and the distribution is more dispersed. Therefore, the ideal funnel diagram is an inverted funnel with large sample studies concentrated at the top and small sample studies scattered at the bottom. If there is publication bias, the funnel plan will be missing corners. And when the circle is out of the funnel, heterogeneity exactly exsits. Therefore, based on the the before-mentioned theory, as can be seen from four graphs in Figure 3, regardless of which analytical dimension of job burnout is focused on, the number of literature for analyzed is enough, no missing corners in the graph, the overall literature also presents certain heterogeneity. While for Figure 3a–d, the publication bias of relevant literature cannot clearly be judged, although the distribution of the cirles is not asymmetric. Therefore, Egger’s regression was continued to be used for the publication bias test [57]. Consequently, this showed that the result for overall burnout (*p* = 0.08 > 0.05) was not significant, the result for emotional exhaustion (*p* = 0.413 > 0.05) was not significant, and the result for personal achievement (*p* = 0.355 > 0.05) was also insignificant. Therefore, no publication bias existed. From the results of Fail-N, we need another 574 articles (much larger than 5n + 10 = 165) to overturn the original results of overall burnout (*n* = 31). Another 392 articles (>5n + 10 = 85) need to be found to override the original result of depersonalization (N = 15). The number of needed pieces of literatures to overturn the results of EE and DP, although small, is also greater than 5n + 10. In general, the degree of publication bias is small.

## 5. Discussion

### 5.1. The Overall Effect Size of the Intervention

From the perspective of overall effect size, intervention has strong effects on both overall effect and depersonalization. The effect on emotional burnout and low personal accomplishment was lower [58,59,60]. This shows that the degree of depersonalization of teachers with job burnout is easier to be relieved and restored after intervention, and the emotional burnout and low sense of accomplishment are more difficult to recover. Additionally, this study also included the results of scales other than the Masrazzi scale when calculating the overall average effect of the intervention on burnout, which may lead to an outcome preference for the overall burnout effect. The effect of intervention on personal achievement is weak, partly due to the lack of relevant literature. On the other hand, it is also possible that personal achievement may be expressed as low or high achievement on different scales. In this study, all statistics of personal achievement are calculated together, which may lead to bias in the results.

### 5.2. Effectiveness of Different Intervention Approaches

In terms of significance and intensity, depersonalization is the easiest in which to intervene, followed by emotional exhaustion and personal achievement. From the significance point of view, although the impact of different interventions on personal achievement is significant, the degree of impact is small. Therefore, emotional exhaustion is more likely to be interfered with than personal achievement. The three dimensions of Masrazzi’s burnout scale increase in severity from emotional exhaustion to depersonalization to personal achievement.

As far as the literature is concerned, the intervention effect of CBI is the most significant, followed by MBI, PT, and EBI. From a theoretical point of view, CBI, as an intervention type, can rely on a variety of specific intervention methods, and MBI can be used as a carrier of CBI; therefore, CBI has a greater possibility of intervention effects. MBI is a widely used intervention method, so there is enough literature to prove the effect of MBI on job burnout. The effect of MBI is sometimes criticized that if MBI is used improperly, there will be side effects [61]. PT, as a vocational training method, can increase teachers’ professional skills, such as teaching, classroom management, and getting along with students, and can be used as a prerequisite to avoid job burnout [62]. However, the actual situation of education and teaching is much more complicated than training, so its intervention effect on job burnout may not be as effective as CBI and MBI However, EBI is a new type of intervention proposed in this study based on previous studies. Due to the unclear definition of the concept and the lack of relevant literature, more comprehensive results cannot be presented. For example, Ghasemi et al. (2023) [19] used a randomized controlled experiment to investigate the effect of group-based cognitive–behavioral intervention on 242 English teachers. Cheek et al. (2003) [21] found that when CBI and music intervention techniques were combined, teachers had lower levels of burnout symptoms than when CBI was used alone. Roeser et al. (2013) [4] investigated the effects of randomization to mindfulness training (MT) or waiting-list control conditions on psychological and physiological indicators of teacher occupational stress and burnout. Chan (2011) [30] investigated the effectiveness of gratitude intervention programs in increasing life satisfaction and reducing symptoms of burnout. In the post-intervention assessment, an increase in life satisfaction and personal fulfillment and a decrease in emotional exhaustion and depersonalization were observed.

In addition, most of the literature in this paper only reported the results of pre-post measurement (*n* = 21) and did not further report the results of follow-up measurement (*n* = 10). Intervention effects usually have a time lag, in which case the actual effects may be ignored, resulting in meta-analysis results that cannot fully reflect the actual effects of intervention.

## 6. Conclusions

The literature mainly shows an increasing trend year by year, and the distribution of the countries studied is relatively average. The school grades of the research objects in the literature and other special characteristics indicated that there is less attention to the intervention of job burnout of teachers in preschool education and higher education, while the intervention of teacher burnout in special education and rural education is also worthy of attention. In addition, this study extracted intervention types from the research literature as well as burnout-related dimensions and their measurement values to conduct a meta-analysis. The results showed that CBI had the best intervention effect on teacher burnout, followed by MBI and PT. At the same time, emotional exhaustion and depersonalization are more susceptible to intervention. For example, Ghasemi et al. (2023) [19] found that the intervention group experienced significantly greater improvements in burnout. Russell et al. (2013) [25] argued that there was no significant increase in mindfulness scores, which means that the mechanism of this reduction in personal burnout still has considerable room for research. Sandilos et al. (2020) [27] found that when teachers participate in children’s social–emotional learning training programs, they will have better interaction with students, and their own job burnout level will decline, while teachers in the control group will have higher job burnout level.

### 6.1. Implications

In practice, schools and all sectors of society should pay more attention to teachers, prevent teacher burnout, and actively intervene in teacher burnout. It is suggested that CBI, MBI, and PT should be combined. In addition, we should strengthen the scientific research of teacher burnout intervention. Teachers are already a special group, but attention should be paid to the more special groups of teachers, such as special education, rural teachers, university, and preschool teachers. For instance, to eliminate teacher burnout, we need to start from many angles. Improving educational environment, adjusting teaching methods, and strengthening communication and interaction are indispensable. Only through comprehensive improvement can teachers’ occupational burnout be effectively alleviated, and teaching effect be improved.

### 6.2. Limitations and Future Study

There are some limitations in this study. For future studies, the sample size could be increased to render the analysis more accurate and comprehensive. Both the accuracy and homogeneity of the classification of intervention remain to be discussed. In addition, this study mainly conducted a meta-analysis of randomized controlled experiments, therefore, in the future, single-group experiments, as well as other experimental types and qualitative studies can be considered to ensure the integrity of research results; besides, this paper did not analyze the mediating influence of intervention duration, time lag, and demographic variables on intervention effect, which also can be explored in future studies. In addition, for future studies, the cognitive–behavioral interventions (CBI), mindfulness-based interventions (MBI), professional training (PT), and emotional-based intervention (EBI) could be assessed and redesigned for different and multiple cultural and various regional backgrounds, especially for rural teachers and ethnic minorities.

## Figures and Tables

**Table 1:** The exclusion criteria.

Order	Exclusion Criteria
Code 1	Not empirical studies (review, protocol)
Code 2	Not on burnout (stress, wellbeing,)
Code 3	Not on teachers (students, parents, physicians, nurse, etc.)
Code 4	Not the effect of intervention (consequence or antecedents of burnout)
Code 5	Not quantitative studies (qualitive studies)
Code 6	Did not report the needed statistical index
Code 7	One-group experiment

**Table 3:** Overall effects of the intervention.

Outcome	*n*	g	SE	Min g	Max g	Z	Q	I^2^
Overall burnout	31	0.644	0.216	0.22	1.068	1.068 **	636.691	95.288
Emotional exhaustion	21	0.387	0.216	−0.036	0.809	1.792 **	337.6	94.076
Depersonalization	13	0.747	0.249	0.259	1.234	3.003 **	156.743	92.344
Personal accomplishment	16	0.147	0.288	−0.419	0.712	0.509 **	292.881	94.878

Note: *n*—the number of included studies; g—the average effect size; SE—standard error of the average effect size; Min/Max g—the minimum and maximum limits of the confidence interval; Z—statistical test used to compute the significance of the average effect size; Q—the statical test used to estimate heterogeneity. ** effect is statistically significant at *p* < 0.01.

**Table 4:** Effectiveness for different types of interventions.

Item	*n*	g	SE	Min g	Max g	Z	Q	I^2^
Emotional exhaustion								
CBI	7	0.876	0.989	−1.062	2.814	0.886 **	305.372	98.035
MBI	10	0.351	0.199	−0.039	0.741	1.765	45.694	80.304
PT	5	0.239	0.131	−0.017	0.496	1.829	7.76	48.451
EBI	2	3.851	3.813	−3.622	11.324	1.01	91.442	98.906
Depersonalization								
CBI	4	2.063	0.765	0.565	3.562	2.698 **	64.142	95.323
MBI	6	0.632	0.299	0.047	1.218	2.117 **	29.66	83.142
PT	3	0.225	0.248	−0.261	0.711	0.908 *	8.539	76.578
EBI	-	-	-	-	-	-	-	-
Personal accomplishment								
CBI	6	−0.205	1.04	−2.244	1.834	−0.197 **	255.509	98.043
MBI	8	0.479	0.332	−0.173	1.13	1.44 **	73.132	90.428
PT	4	0.140	0.195	−0.243	0.522	0.714 *	10.214	70.629
EBI	2	3.851	3.813	−3.622	11.324	1.01	91.442	98.906

Note: *n*—the number of included studies; g—the average effect size; SE—standard error of the average effect size; Min/Max g—the minimum and maximum limits of the confidence interval; Z—statistical test used to compute the significance of the average effect size; Q—the statical test used to estimate heterogeneity. * Effect is statistically significant at *p* < 0.05; ** effect is statistically significant at *p* < 0.01.

## Data Availability

The datasets generated and/or analysed during the current study are available from the corresponding author upon reasonable request.
